# IL-17A and IL-2-Expanded Regulatory T Cells Cooperate to Inhibit Th1-Mediated Rejection of MHC II Disparate Skin Grafts

**DOI:** 10.1371/journal.pone.0076040

**Published:** 2013-10-11

**Authors:** Benoît Vokaer, Louis-Marie Charbonnier, Philippe H. Lemaître, Chloé Spilleboudt, Alain Le Moine

**Affiliations:** Institute for Medical Immunology, Université Libre de Bruxelles, Gosselies, Belgium; Centre Hospitalier Universitaire Vaudois (CHUV), Université de Lausanne, Switzerland

## Abstract

Several evidences suggest that regulatory T cells (Treg) promote Th17 differentiation. Based on this hypothesis, we tested the effect of IL-17A neutralization in a model of skin transplantation in which long-term graft survival depends on a strong in vivo Treg expansion induced by transient exogenous IL-2 administration. As expected, IL-2 supplementation prevented rejection of MHC class II disparate skin allografts but, surprisingly, not in IL-17A-deficient recipients. We attested that IL-17A was not required for IL-2-mediated Treg expansion, intragraft recruitment or suppressive capacities. Instead, IL-17A prevented allograft rejection by inhibiting Th1 alloreactivity independently of Tregs. Indeed, T-bet expression of naive alloreactive CD4+ T cells and the subsequent Th1 immune response was significantly enhanced in IL-17A deficient mice. Our results illustrate for the first time a protective role of IL-17A in CD4+-mediated allograft rejection process.

## Introduction

We and others recently identified a role for IL-17A producing CD4+ and CD8+ T cells in the allograft rejection process [1]–[3]. Indeed, IL-17A neutralization has been shown to prolong allograft survival in either minor mismatched skin transplants [2] or in T-bet deficient recipients grafted with MHC-mismatched cardiac allografts [1], [3]. However, single IL-17A deficiency is no longer effective to prevent spontaneous rejection of MHC I and/or MHC II mismatched skin allografts in untouched recipients. This probably reflects the redundant pathways of allograft rejection involving numerous alloreactive effector cells belonging to T helper (Th)1, Th2 and CD8+ T cells [4]. Moreover, in agreement with the concept of mutual regulation of Th subsets, early production of IFN-γ or IL-4 from differentiating Th0 cells potentially impairs development of alloreactive IL-17A producing T cells [5].

By inhibiting Th1 and Th2 cells, Tregs may promote de novo Th17 differentiation as shown in different experimental models [5], [6]. On the other hand, in vivo Treg expansion by supplying exogenous IL-2 is an efficient strategy to dampen autoimmunity and transplant rejection [7]–[9]. In mice, this goal was achieved by coupling IL-2 to an anti-IL-2 monoclonal antibody that targets specifically Treg cells [9], [10]. By using this approach, we hypothesized that a Treg boost might promote an IL-17A-dependent mechanism of graft rejection by inhibiting the Th1 and Th2 pathways in a well-established murine model of CD4+ T cell-mediated alloreactivity. Surprisingly, our results indicated that IL-17A has the opposite effect by supporting long-term acceptance induced by the IL-2-mediated Treg expansion in vivo. We further demonstrated that IL-17A directly inhibits Th1 alloreactive cells and cooperates with IL-2-expanded Tregs for preventing graft rejection.

## Materials and Methods

### Mice

C57Bl/6.C-H-2bm12 mice (bm12) were obtained from The Jackson Laboratory. Wild type C57Bl/6 (B6) mice were purchased from Harlan, Netherlands. IL-17A-/- C57Bl/6 mice were kindly provided by Dr Iwakura. Eight to twelve weeks old animals were used and animals were bred in our specific pathogen-free animal facility. All animals received humane care in compliance with the Principles of Laboratory Animal Care formulated by the National Institute of Health (Guide for the Care and Use of Laboratory Animals, Eighth Edition, National Research Council, 2010) and protocols were approved by the Ethical Committee from the Biopole ULB Charleroi, Université Libre de Bruxelles (agreement # LA2500519).

### Skin grafting, treatments with antibodies and IL-2/anti-IL2 complex

Mice were anaesthetized with a mixture of xylazine (Rompun) 5% and ketamine 10% in phosphate-buffered saline (PBS). A total of 100 µL per 20g of body weight was injected intraperitoneally. Skin grafting was performed according to an adaptation of the method of Billingham and Medawar. Briefly, skin grafting was conducted by grafting full-thickness tail skin (1 cm^2^) on the lateral flank. Grafts were monitored daily after the removal of the bandage on day 8 and considered rejected when more than 75% of epithelial breakdown had occurred. IL-2/anti-IL-2 complex was formed by incubating 1 µg recombinant mouse IL-2 (eBioscience) and 9 µg of purified anti-mouse IL-2 (clone JES6-1A12) (Bio-X-cell) for 30 min at 37°C. This complex was administered at days 0, 1 and 2 after transplantation. The neutralizing anti-IL-17A monoclonal antibody (clone MM17F3, provided by Dr Huytenhove Catherine, Université Catholique de Louvain, Belgium) and the Isotype control antibody (clone MOPC-21, obtained from BioXcell) were intraperitoneally injected at a dose of 400 µg twice a week after transplantation.

### RNA extraction and real-time RT-PCR

Total RNA was extracted from skin grafts using the MagnaPure LC RNA Isolation Kit III for tissue (Roche Diagnostics). Reverse transcription and real-time PCR were performed using LightCycler-RNA Master Hybridization Probes (one-step procedure) on a Lightcycler apparatus (Roche Diagnostics). Number of mRNA copies was evaluated by using standard curve for each gene of interest and was normalized to β-actin as housekeeping gene. Primer and probe sequences were as following: for β-actin: forward CCGAAGCGGACTACTATGCTA, reverse TTTCTCATAGATGGCGTTGTTG, probe ATCGGTGGCTCCATCCTGGC; for IFN-γ: forward GGATGCATTCATGAGTATTGC, reverse GCTTCCTGAGGCTGGATTC, probe TTTGAGGTCAACAACCCACAGGTCCA; for CXCL9: forward GAACCCTAGTGATAAGGAATGCA, reverse CTGTTTGAGGTCTTTGAGGGATT, probe CATCAGCACCAGCCGAGGCACG; for CXCL10: forward GCCGTCATTTTCTGCCTCAT, reverse GCTTCCCTATGGCCCTCATT, probe TCTCGCAAGGACGGTCCGCTG.

### Flow Cytometry

Pacific-Blue- (PB), Allophycocyanin- (APC) or BD HorizonTM V450- (V450) conjugated anti-mouse CD4 (clone RM4-5), APC-conjugated anti-mouse CD25 (clone PC61 and 7D4), Phycoerythrin (PE) or biotin-conjugated anti-mouse ICOS (clone 7E.17G9), PE-conjugated anti-mouse CTLA4 (clone UC10-4F10-11), APC-conjugated anti-IL-17A (clone B56), PE-conjugated anti-mouse IFN-γ (clone XMG1.2), and anti-mouse CD16/CD32 (Fc block, clone 2.4G2) monoclonal antibodies and PercP-conjugated streptavidin were purchased from BD Pharmingen. PB-conjugated anti-mouse Foxp3 (clone FJK-16s) and PE-Cyanin7-conjugated anti-mouse CD39 (clone 24DMS1) were purchased from eBioscience. APC-conjugated anti-mouse LAP (membrane bound TGF-β) (clone TW7-16B7) was purchased from Biolegend. Flow cytometry analyses were performed on a CyAn-LX cytometer using Summit 4.1 software (DakoCytomation). Cell suspensions were incubated for 10 minutes with Fc block, stained for surface markers for 20 minutes, washed with Phosphate Buffer Saline/Bovine Serum Albumin 0.1%/NaN_3_0.01%. Foxp3 and T-bet stainings were performed after cell surface marker labeling by using eBioscience Fixation/Permeabilization and Permeabilization buffers according to the manufacturer's instructions. To isolate graft infiltrating lymphocytes, skin grafts were minced and incubated at 37°C in a phosphate buffered solution containing 2.5 mg/mL of type I collagenase (Sigma) and 0.25 mg/mL hyaluronidase (Sigma) for 2 hours. BD FACSTM Lysing Solution was used for lysing red blood cells after immunofluorescence staining according to the manufacturer's instructions for peripheral blood mononuclear cells (PBMCs) samples.

### Mixed lymphocyte culture, in vitro suppression assay and cytokine detection

For mixed lymphocyte cultures (MLC), responder cells (2.5×10^6^ cells/mL) isolated from draining lymph nodes (inguinal and axillary) were stimulated with irradiated splenocytes (2,000 Rad; 2.5×10^6^ cells/mL) from either B6 (syngeneic), bm12 (allogeneic) in 48-wells flat-bottom plates (Nunc, 150687, Roskilde, Denmark). Cultures were incubated at 37°C in a 5% CO2 atmosphere in medium consisted of RPMI 1640 medium supplemented with 2 mM L-glutamine, 1 mM nonessential amino acids, 5% heat-inactivated fetal calf serum (FCS) and 1 mM sodium pyruvate. IFN-γ and IL-13 production was measured in culture supernatants after 48 hours and 72 hours respectively.

For in vitro suppression assay, WT CD4+ T cells were isolated using CD4 negative isolation kit from Miltenyi, and used as responder cells. Treg cells were defined as CD4+CD25^high^ cells isolated with a MOFLO cell sorter and used as suppressor cells. These Tregs were purified from WT or IL-17A-/- mice previously treated with the IL-2/anti-IL-2 complex during 3 consecutive days, and were sorted on day 5, which corresponds to the peak of Treg expansion. Responders were used at a fixed concentration of 10^5^ cells per well and stimulated with 2 µg/mL of soluble anti-CD3 antibody (145-2C11) in the presence of 4.10^5^ RAG-/- γc-/- spleen cells used as feeder cells. Different numbers of Tregs were added in order to have various ratios of responders/suppressors. Cells were set up as in standard 96-well proliferation assays in triplicate. The IFN-γ production was analysed at day 3.

Concerning the MLC with IL-17A supplementation, naive CD4+ T cells from B6 mice (responders) were magnetically purified with a naive CD4+CD62L+ T cell Isolation Kit II from Miltenyi Biotec (#130-093-227). Stimulator cells are T cell-depleted spleen cells from either B6 or bm12 mice negatively sorted by exclusion of CD90+ cells, using the mouse CD90.2 microbeads kit from Miltenyi Biotec (#130-049-101). All magnetic cell purifications were performed according to the manufacturer's protocol. Then, cells were seeded in a 96-well culture plate in triplicate. Mouse IL-17A recombinant protein was purchased from eBioscience. IFN-γ was detected in culture supernatants by ELISA according to the manufacturer instruction (R&D, DUOSET® IFN-γ detection kit). The detection threshold was 30 pg/ml.

### Statistical analyses

Statistical analyses of differences between groups were performed using the two-tailed Mann-Whitney nonparametric test. Results are expressed as mean ± SEM. Graft survival curves were compared by the log-rank test. p<0.05 is considered statistically significant.

## Results

### IL2/anti-IL2 complex fails to delay skin allograft rejection in IL-17A deficient mice

**Figure 1:**
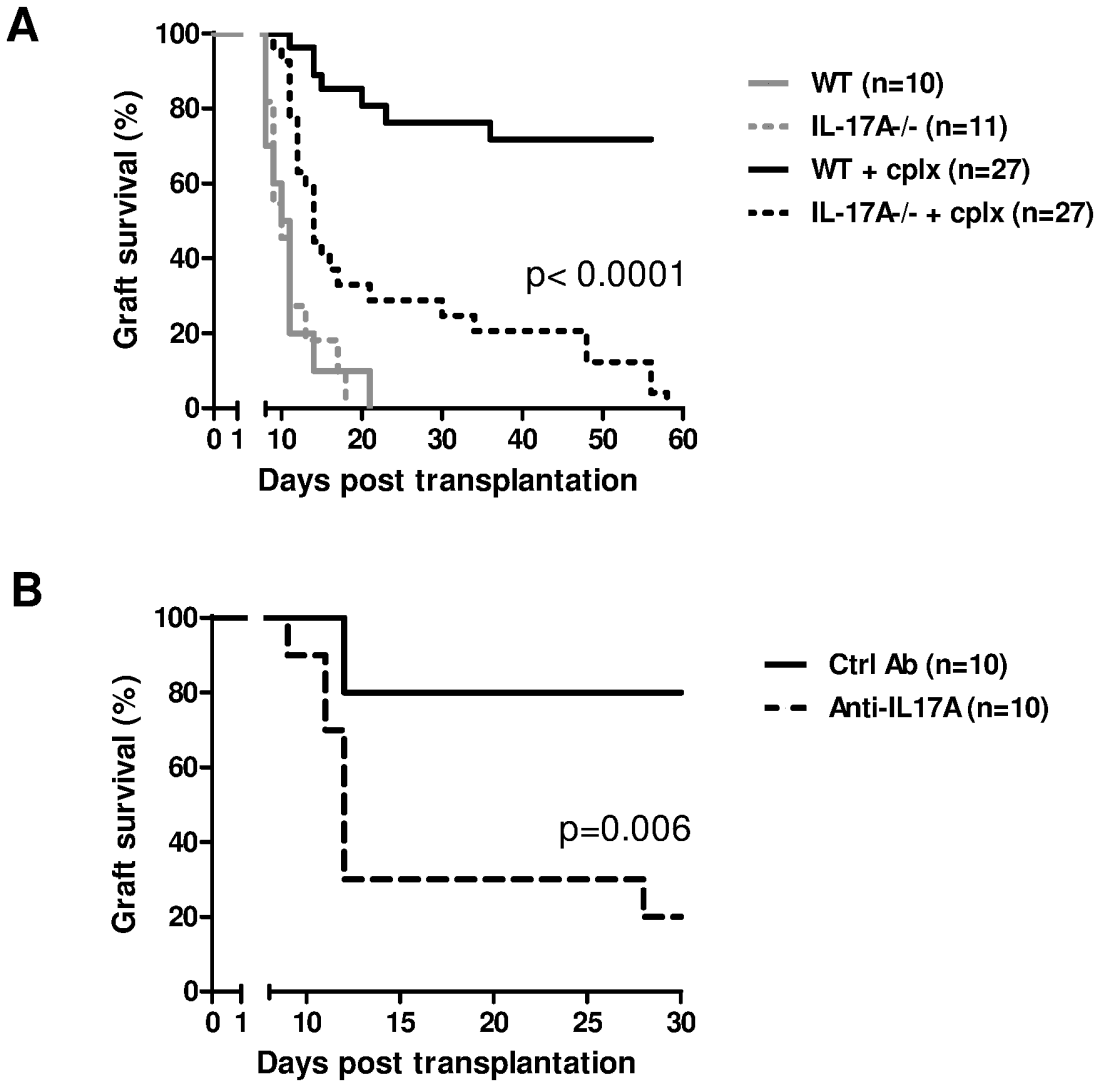
IL-17A is required for long-term allograft acceptance mediated by Tregs in the context of a single MHC class II disparity. (**A**) bm12 skins were transplanted on either wild type (WT) or IL-17A-/- B6 recipients that were treated or not with the IL-2/anti-IL-2 complex (Cplx) as described in the method. Graft survivals were compared. Cumulative data of five independent experiments are shown. (**B**) bm12 skins were transplanted on IL-2/anti-IL-2 treated B6 recipients injected with an anti-IL-17A mAbs or with purified rat IgG control antibody. Cumulative data of two independent experiments are shown.

In order to study the role of IL-17A in the context of a Treg-mediated dampening of alloreactivity, we took advantage of the skin allograft rejection model across a single MHC II disparity. In this combination, IL-2/anti-IL-2 treatment strongly prolongs skin allograft survival through Treg expansion [11]. Tail skins from bm12 donors were transplanted on either B6 wild type (WT) or IL-17A-/- recipients, treated or not with the IL-2/anti-IL-2 complex. As expected, acute rejection occurred as soon as day 10 in all WT and IL-17A-/- untreated recipients. In IL-2/anti-IL-2 treated recipients, whereas almost 80% of WT recipients retained their graft for more than 60 days, the IL-17A recipient deficiency restored rejection with a median survival time of 15 days (**fig. 1A**). Similarly, IL-17A blockade with a monoclonal antibody also restored rejection in wild type recipients treated with the IL-2/anti-IL-2 complex, confirming a protective effect mediated by IL-17A (**fig. 1B**).

### IL-17A is not required for IL-2/ anti-IL-2-induced Treg expansion

**Figure 2:**
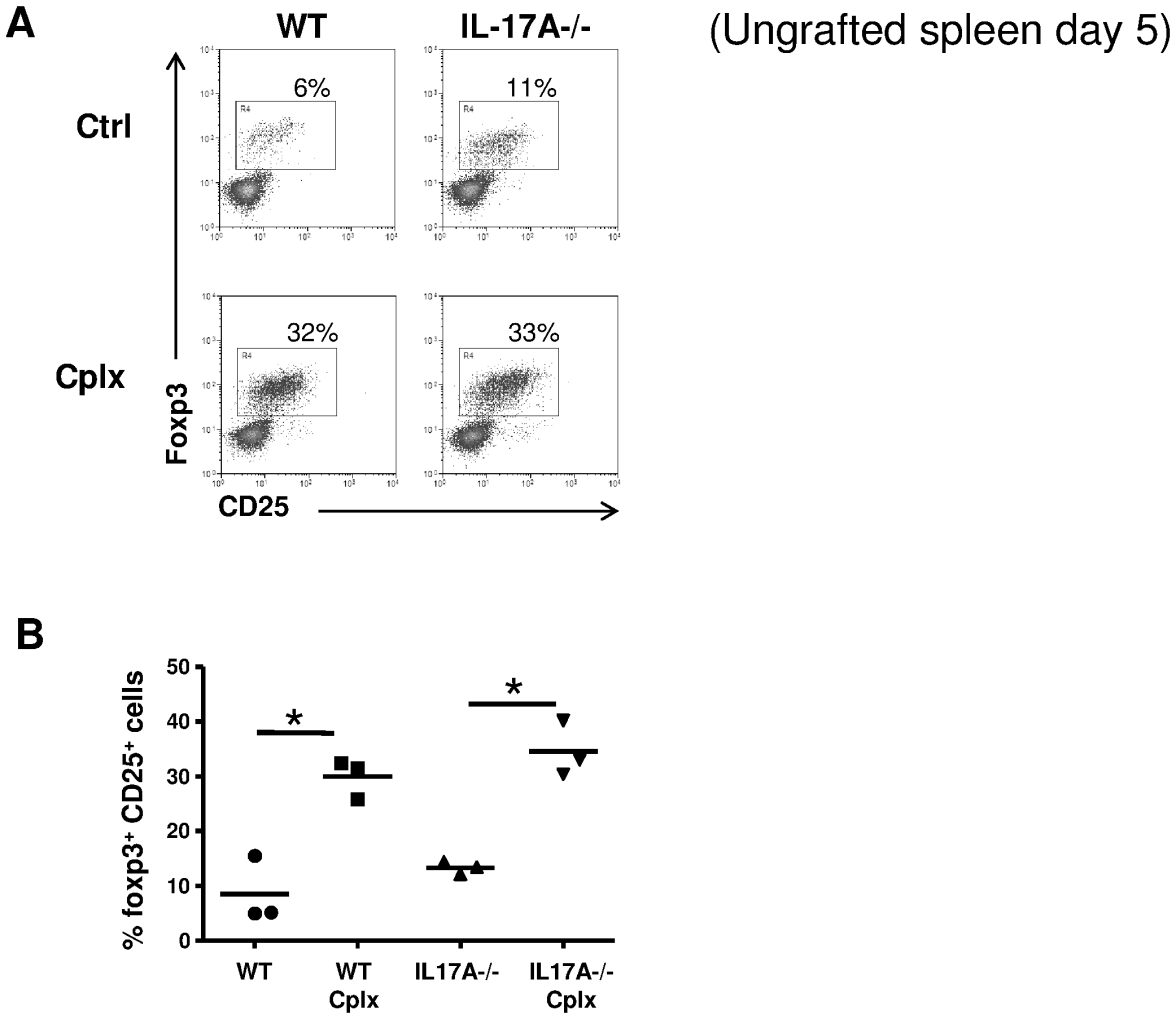
IL-17A deficiency does not affect IL-2/anti-IL2-mediated Treg expansion. Wild type (WT) or IL-17A-/- B6 mice were treated or not with the IL-2/anti-IL-2 complex (Cplx). Spleen cells were isolated 5 days later. Foxp3 and CD25 expression by CD4+ T cells is shown. (**A**) Representative dot plots and (**B**) a pool of individual mice are shown (n = 3/group). The mean is represented (*p<0.05) and data are representative of two independent experiments. Similar results were obtained in bm12 grafted animals.

We further determined whether the absence of IL-17A could affect the IL-2/anti-IL-2-mediated Treg expansion. Wild type and IL-17A-/- ungrafted mice were injected with the IL-2/anti-IL-2 complex and spleen cells were harvested at day 5, corresponding to the peak of Treg expansion [9], [11]. Around 10% of CD25+Foxp3+ cells among CD4+ T cells were found in untreated groups (WT and IL-17A-/-) whereas more than 30% of Tregs were measured in both WT and IL-17A-/- IL-2/anti-IL-2-treated mice (**fig. 2A and 2B**). Because ungrafted mice may not be representative of conditions with ongoing alloresponses and non-specific inflammation related to the transplant procedure, this experiment was repeated in bm12 grafted animals and showed similar results (data not shown).

### IL-17A deficiency does not impair Treg recruitment and suppressive capacities

**Figure 3:**
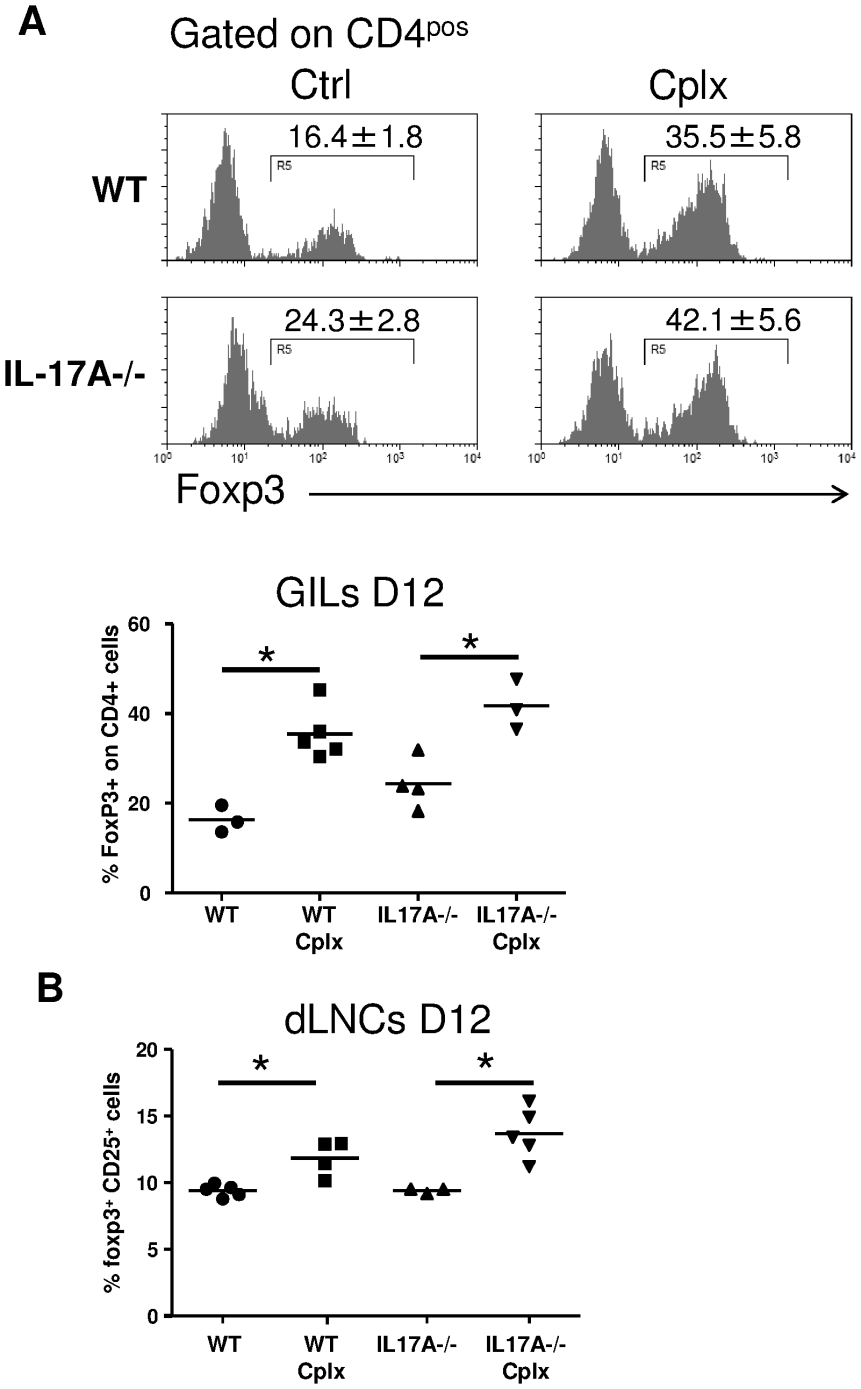
Intragraft Treg recruitment does not require IL-17A. Wild type (WT) or IL-17A-/- B6 mice were grafted with a bm12 skin and treated or not with the IL-2/anti-IL-2 complex (Cplx). At day 12 post-transplantation, grafts and draining lymph nodes were harvested for flow cytometry analysis. (**A**) Expression of Foxp3 among CD90+ CD4+ graft infiltrating lymphocytes (GILs). Histograms of GILs and a pool of individual mice are shown (n = 3–5/group). (**B**) Expression of CD25 and Foxp3 by CD4+ draining lymph nodes cells is shown (n = 3–5/group). The mean is represented and data are representative of two independent experiments (*p<0.05).

Next, we assessed whether IL-17A deficiency could impair the recruitment or function of IL-2-expanded Tregs. Wild type and IL-17A-/- B6 recipients were grafted with a bm12 skin and treated or not with the IL-2/anti-IL-2 complex. At day 12, flow cytometry analysis of both graft infiltrating lymphocytes (GILs) and graft-draining lymph node T-cells was performed. Similar percentages of infiltrating Tregs were observed in WT and IL-17A-/- recipients and this was true for both the control and the IL-2/anti-IL-2 treated groups (**fig. 3A and 3B**).

**Figure 4:**
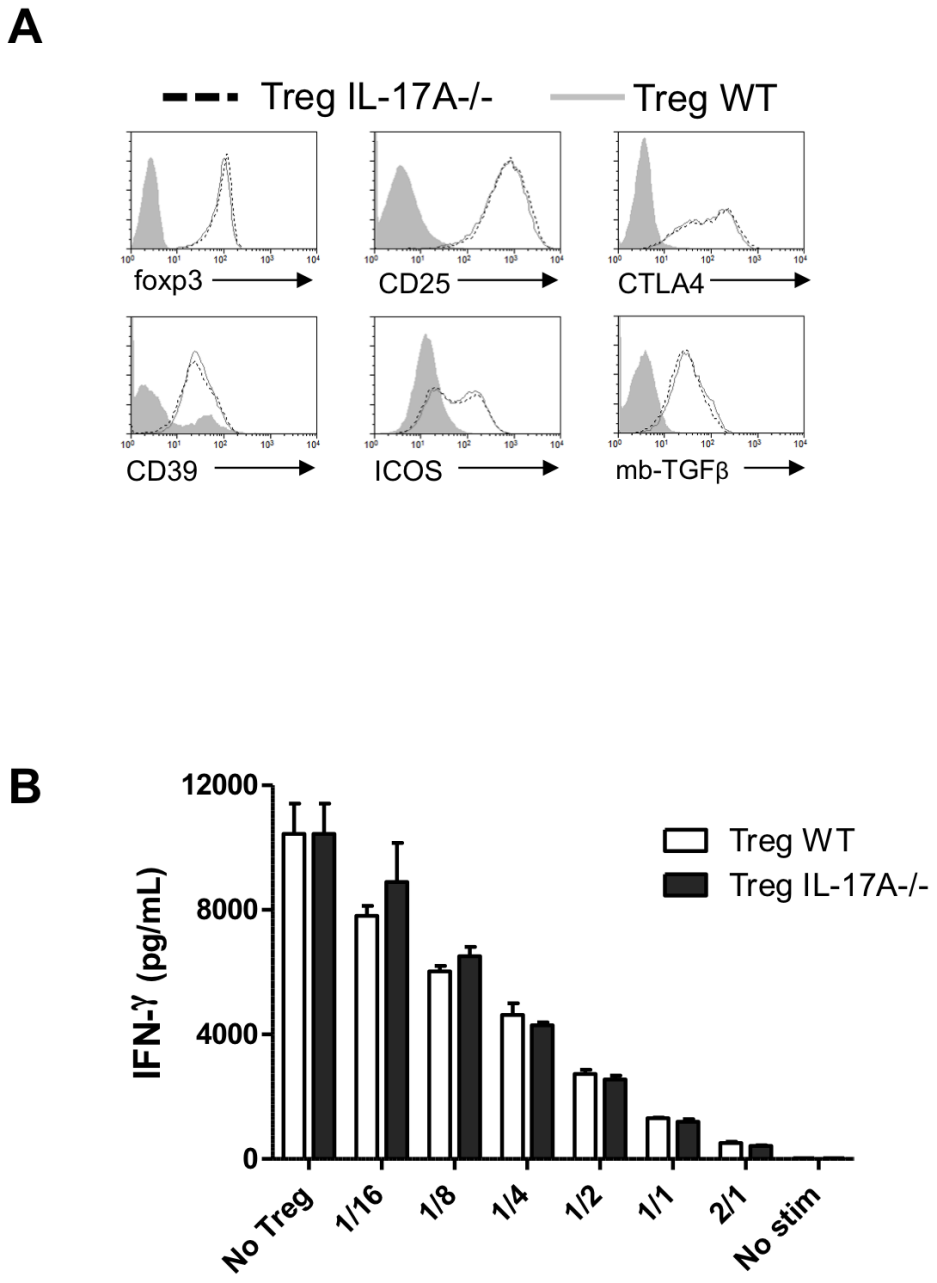
Suppressive capacities of IL-2-expanded Tregs are not affected by IL-17A deprivation. (**A**) The phenotype of splenic IL-2/anti-IL-2-expanded Tregs (defined as CD4+ Foxp3+ cells) from WT or IL-17A-/- mice. Flow cytometry analysis was performed at day 5 after treatment with respect to the expression of Foxp3, CD25, CTLA4, CD39, ICOS and membrane-bound TGF-β. Histogram of one representative mouse of three is represented and grey curve represents expression in CD4+ effector cells. (**B**) In vitro suppression of WT CD4+ T cells (described as Teff) by various numbers of CD4+CD25high Treg cells (Treg) isolated from WT and IL-17A-/- mice treated with the Il-2/anti-IL-2 complex. The IFN- γ production of WT CD4+ T cells after polyclonal stimulation with anti-CD3 monoclonal antibodies is shown. The results are expressed as mean ± SEM of triplicates. In all panels, one representative experiment of two is shown.

Regarding IL-2-expanded Treg suppressive capacities, the expression of several molecules involved in Treg functions was studied in IL-2/anti-IL-2-treated WT or IL-17A-/- mice. IL-2/anti-IL-2-expanded Tregs from IL-17A-/- Tregs showed similar expression of Foxp3, CD25, CTLA4, CD39, ICOS and membrane bound TGF-β (mbTGF-β), in comparison to WT Tregs (**fig. 4A**). Then, we characterized the influence of IL-17A-/- Treg suppressive capacities ex-vivo. For this purpose, IFN-γ production by WT CD4+ T cell was measured after anti-CD3 stimulation in the presence of increased ratios of Tregs harvested from respectively IL-2/anti-IL-2-treated WT or IL-17A deficient animals. In accordance with their similar Treg phenotype, IL-2-expanded Tregs from IL-17A-/- and WT mice exhibited comparable suppressive activity in vitro (**fig. 4B**).

### Th1 alloreactivity is exacerbated in IL-17A deficient recipients despite Treg expansion

**Figure 5:**
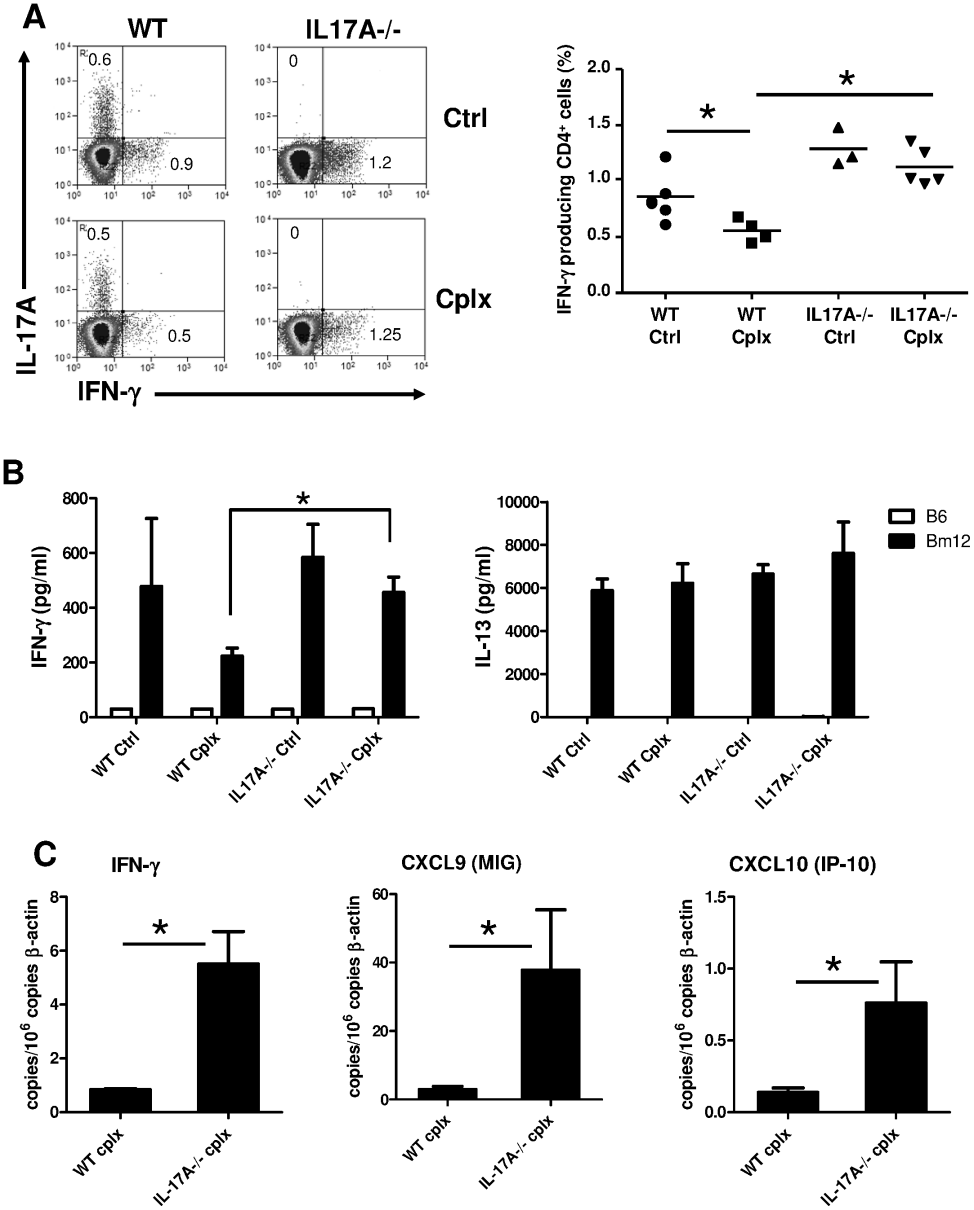
Th1 immune response is exacerbated in IL17A deficient recipients despite IL-2-mediated Treg expansion. Skins from bm12 donors were transplanted on either WT or IL-17A-/- B6 recipients that were treated or not with the IL-2/anti-IL-2 complex. Grafts and draining lymph nodes cells were harvested at day 12 post transplantation. (**A**) Representative plots of IL-17A and IFN-γ expression by CD3+CD4+ draining lymph node cells after 4 hours stimulation with PMA and ionomycin. The mean is represented (n = 3–5 mice/group). (**B**) Draining lymph node cells (2×10^6^/mL) from recipients in each group were stimulated with either B6 (Syngeneic) or bm12 (Allogeneic) irradiated spleen cells (2.5×10^6^/mL). IFN-γ an IL-13 production was quantified (n = 4–9/group). Results are expressed as mean ± SEM. (*p<0.05). (**C**) Intragraft mRNA expression of Th1 related gene was measured in each group (n = 4/group) and normalized to the expression of β-actin. Data are representative of two independent experiments.

Since IL-17A does not affect development, recruitment and function of IL-2-expanded Tregs, we further hypothesized a direct effect of IL-17A on alloreactive CD4+ T helper cells that mediate allograft rejection. WT and IL-17A-/- recipients were grafted with a bm12 skin and treated or not with the IL-2/anti-IL-2 complex. Twelve days later, graft draining lymph nodes and bm12 grafts were harvested to investigate cytokine and chemokine productions. Whether or not transplanted animals received exogenous IL-2, IL-17A-/- recipients displayed a two-fold higher frequency of IFN-γ producing CD4+ T cells among draining lymph nodes compared to WT (**fig. 5A**). Next, we assessed the cytokine production by recipient-derived CD4+ T cells in response to the bm12 alloantigen. Draining lymph node cells from all groups were cultured with either B6 (syngeneic) or allogeneic bm12 (donor) irradiated spleen cells. As shown in **figure 5B**, allogeneic bm12 (donor) stimulation (but not syngeneic) triggered an IFN-γ production that was downregulated by an in vivo IL-2/anti IL-2 treatment in WT animals. On the contrary, IFN-γ production by IL-17A-/- T cells remained high whether or not the recipient was treated with complexed IL-2. Interestingly, production of IL-13 in response to bm12 antigen was affected neither by previous IL-2/anti-IL-2 treatment nor by IL-17A deficiency. Analysis of intragraft mRNA expression revealed increased amounts of IFN-γ and Th1-associated chemokines (CXCL9, CXCL10) in bm12 graft harvested from IL-17A-/- recipients compared with WT recipients (**fig. 5C**).

### IL-17A inhibits T-bet expression in Th1 alloreactive cells

**Figure 6:**
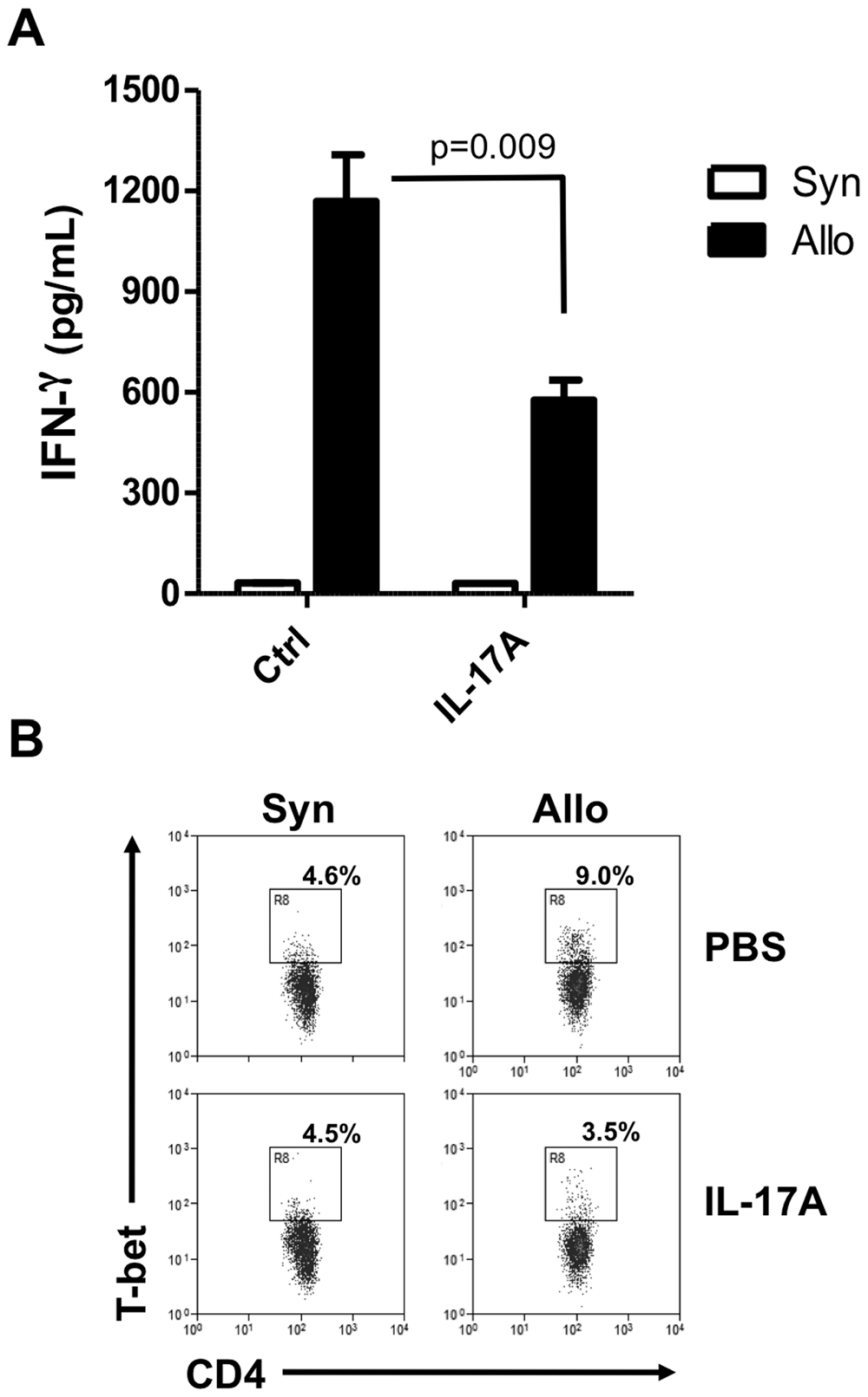
IL-17A inhibits Th1 differentiation by preventing T-bet expression. Naive CD4+ T cells (10^5^) were sorted from B6 mice and stimulated for 96 hours with either syngeneic (B6) or allogeneic (bm12) T cell depleted spleen cells (4.10^5^). When indicated, recombinant mouse IL-17A was added in culture wells at 40 ng/ml. (**A**) The IFN-γ concentration was measured in supernatants by ELISA and the mean ± SEM of quintuplicates are shown. One representative experiment of two is shown (**B**) The percentages of T-bet+ cells among responder CD4+ T cells were determined by intracellular staining. The mean of triplicates is represented and one representative experiment of two is shown.

Interleukin-17A has been shown to prevent Th1 immune response in the CD45RB transfer model of colitis [12]. Based on this observation, we could hypothesize that IL-17A directly inhibits alloreactive Th1 de novo differentiation in our model. To test this hypothesis, B6 naive CD4+ T cells were purified and stimulated with either bm12 or B6 antigen presenting cells in the presence or not of recombinant IL-17A. In this condition, not involving Treg cells, allogeneic bm12 stimulation induced IFN-γ production that was decreased in the presence of IL-17A (**fig. 6A**). Concomitantly, T-bet expression by naive CD4+ T cells was up-regulated after bm12 stimulation but returned to background levels after adding IL-17A in the culture medium (**fig. 6B**).

## Discussion

In contradiction with many studies reporting a deleterious pro-inflammatory effect of IL-17A (including our own), herein we report a protective role of this cytokine. This effect seems to ensue from a synergy between Treg and IL-17A in the inhibition of a Th1 pathway of allograft rejection. Although IL-17A can be easily detected in draining lymph nodes, WT recipients rapidly reject bm12 skin allografts. In this condition, a putative protective role of this cytokine remains undetectable due to a too rapid kinetic of rejection. In contrast, a protective effect becomes detectable when Tregs dampen allograft rejection. We initially postulated that IL-17A deficiency could interfere directly or indirectly with Treg expansion and/or function since our model of graft acceptance is dependent on Treg homeostasis [11]. However, we found similar expression of key immunosuppressive molecules expressed by both IL-17A-/- and WT Tregs. Accordingly, in vitro anti-CD3 stimulation assays confirmed their comparable ability to suppress a Th1 immune response. These results appear in contradiction with previous works showing that Tregs require IL-17A for mediating contact-dependent T cell suppression [13], [14]. In these experiments, Tregs expressed lower levels of suppressive molecules such as mTGF-β, Foxp3, CTLA-4 and GITR. The fact that we studied the role of IL-17A in the context of IL-2/anti-IL-2-expanded Tregs endowed with enhanced suppressive capacities, may explain this apparent discrepancy [9].

IL-17A has been described to promote Treg recruitment in tumor microenvironment [14]. This effect involves the expression of several IL-17A-induced adhesion molecules (CCL17, CCL22). Although we did not assess directly this issue, we found similar percentages of Tregs in allografts and draining lymph nodes from both IL-17A-/- and WT recipients. This reasonably precludes the possibility that accelerated allograft rejection in IL-2-treated IL-17A-/- recipients was due to a defect in Treg recruitment.

Since IL-17A deficiency did not affect Tregs themselves, we further hypothesized a direct inhibitory effect of IL-17A on alloreactive CD4+ effector T cells. This was supported by the increased IFN-γ production observed in graft and draining lymph nodes (dLN) of IL-17A-/- recipients, independently of a comparable IL-2-mediated Treg expansion. Consequently, elevated amounts of mRNAs coding for CXCL9 and CXC10, two typical IFN-γ-induced chemokines, were detected in IL-17A-/- recipients. Furthermore, mixed lymphocyte cultures using dLN cells and bm12 stimulator cells demonstrated the inhibitory effect of IL-17A on anti-donor Th1 cells. Although both Th1 and Th2 cytokines have been described to promote allograft rejection in the bm12 to B6 combination [4], [15], [16], we assume that the restored allograft rejection in IL-17A-/- recipients resulted from an exacerbated Th1 pathway. This is supported by three arguments. First, WT and IL-17-/- dLN cells produced comparable amounts of IL-13 (a Th2 cytokine) in mixed lymphocyte culture with donor antigens, suggesting that Th2 cells are not affected by IL-17A. Second, IFN-γ plays an indispensable role in this particular transplant setting by up-regulating the expression of the single MHC class II alloantigen on donor keratinocytes [16]. Finally, CXCL9 and CXCL10, two chemokines induced by IFN-γ are required for tissue damages in this allogeneic combination [17].

The clinical relevance of our experimental model using a single MHC II disparity might appear limited. Nevertheless, it allows a meaningful mechanistic experimental approach for several reasons. By keeping CD8+ T cell alloreactivity out, this CD4+ T cell-restricted model allows us to investigate the reciprocal interplay between Th1, Th17 and Treg cells in the context of transplantation. Indeed, CD8+ alloreactive T cells that directly recognize allogeneic MHC class I on donor dendritic cells provide an early and abundant source of IFN-γ, which constitutes a barrier for Th17 immune responses [4], [5]. In order to translate our findings in the context of other transplant antigen disparities, we tested the IL-2/anti-IL-2 complex in the same skin graft model using full MHC mismatched (Balb/C into B6) and multiple minor mismatched grafts (Sv129.B6 into C57Bl/6). In these settings, the IL-2/anti-IL-2 complex failed to delay graft rejections that occurred as soon as day 14 in both cases (**fig. S1**). This probably results from a larger alloreactive repertoire, which escapes to suppression by IL-2-expanded Tregs. Consequently, we were not able to demonstrate a faster rejection in IL-17A-/- recipients.

In another context, O'connor et al. described a similar regulatory role of IL-17A in a model of Th1-mediated colitis induced by the transfer of purified CD4+ effector T cells in lymphopenic hosts [12]. In this system, IL-17A also inhibited the Th1 lineage independently of Tregs. Consistently with this, we showed that the addition of IL-17A to naive T helper cells directly inhibited their IFN-γ production in mixed lymphocyte culture. Moreover, we observed that IL-17A dampens Th1 cells, independently of Tregs, through the inhibition of T-bet expression, the key transcription factor for Th1 differentiation. Several studies reported the inhibition of IL-12 responsiveness by IL-17A during Th1 differentiation either through IL-12Rβ2 downregulation in human PBMC [18], [19] or downstream STAT4 phosphorylation [12]. Therefore, it seems likely that IL-17A-mediated Th1 inhibition requires systems in which APC produce IL-12. This is in agreement with what we describe in **figure 6** in which naive CD4+ T cells are stimulated with non-irradiated donor splenocytes still able to produce IL-12. In contrast, no difference in term of IFN-γ production was observed after polyclonal stimulation with anti-CD3 antibody (**fig. 4B**) in the absence of APC and IL-12.

Regarding the induction of long-term graft acceptance, the use of the IL-2/anti-IL-2 complex represents an elegant model to study the impact of IL-17A on Th1 cells in vivo, independently of Tregs. Indeed, by choosing a particular anti-IL-2 monoclonal antibody (clone JES6-1A12), we were able to expand specifically the CD25+ regulatory T cell subset without interacting directly with CD4+ effector cells [10]. In contrast, tolerance induction protocols, that use costimulatory blockade and/or donor specific transfusion, also inhibit the allogeneic CD4+ effector cells by preventing their full activation.

Dendritic cells (DC) also express the IL-17RA. Therefore, it is possible that IL-17A inhibits allograft rejection in our model by regulating allogeneic DC functions. This hypothesis is unlikely since several studies demonstrated that IL-17A promoted the maturation of DC progenitors by increasing cell surface costimulatory molecules (CD40, CD80 and CD86) and MHC class II molecules [20].

In summary, although it is generally considered that IL-17A neutralization prevents inflammation and autoimmunity, we demonstrated a protective role of this cytokine by Th1-Th17 reciprocal regulation. This contributes with IL-2 expanded Tregs to ensure long-term allograft survival.

## Supporting Information

Figure S1 **L-2/anti-IL-2 complex does not prevent graft rejection in the context of multiple minor antigens or full MHC disparity.** (**A**) Sv129.B6 skins were transplanted on either wild type (WT) or IL-17A-/- (KO) B6 recipients that were treated or not with the IL-2/anti-IL-2 complex (Cplx) as described in the method. (**B**) Balb/c skins were transplanted on B6 recipients (WT) injected or not with the IL-2/anti-IL-2 complex (Cplx). Graft survivals were compared using the log rank test.
(TIF)
