# Supplementary figures and images for: IL-17A and IL-2-Expanded Regulatory T Cells Cooperate to Inhibit Th1-Mediated Rejection of MHC II Disparate Skin Grafts
Source: PLoS One. 2013 Oct 11;8(10):e76040. doi: 10.1371/journal.pone.0076040 (PMC3795694; doi:10.1371/journal.pone.0076040)

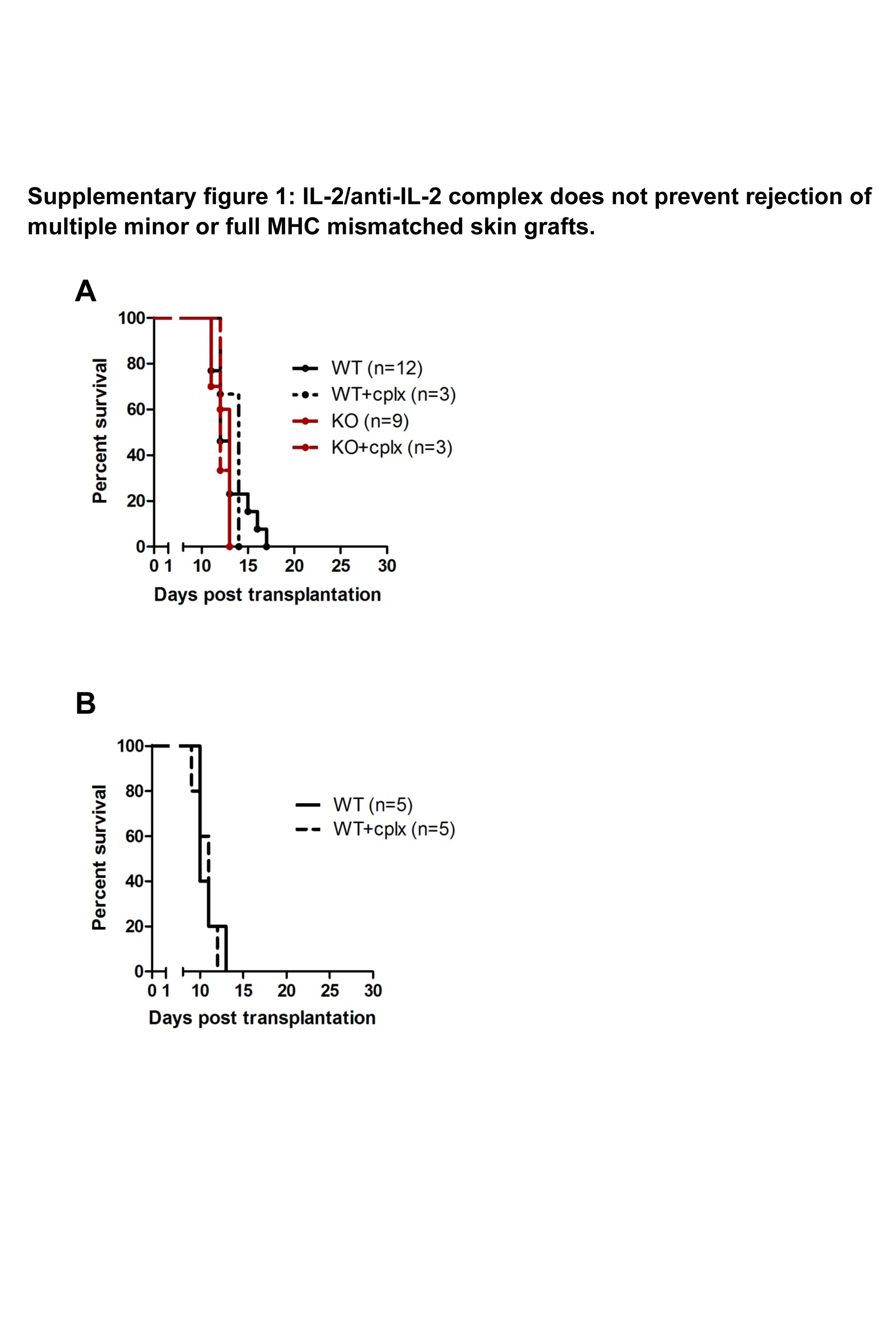

Supplement: Figure S1 — L-2/anti-IL-2 complex does not prevent graft rejection in the context of multiple minor antigens or full MHC disparity. (A) Sv129.B6 skins were transplanted on either wild type (WT) or IL-17A-/- (KO) B6 recipients that were treated or not with the IL-2/anti-IL-2 complex (Cplx) as described in the method. (B) Balb/c skins were transplanted on B6 recipients (WT) injected or not with the IL-2/anti-IL-2 complex (Cplx). Graft survivals were compared using the log rank test. (TIF) [file pone.0076040.s001.tif]
